# Spinopelvic compensation and functional outcomes after short-segment lumbar fusion in patients with residual sagittal imbalance

**DOI:** 10.3389/fsurg.2026.1928933

**Published:** 2026-09-28

**Authors:** Zehua Jiang, Haojun Cui, Xusheng Bi, Xin Tan, Yaohui Qiu, Wenjun Du, Hui Li, Rusen Zhu

**Affiliations:** 1 Tianjin Union Medical Center, Tianjin Medical University, Tianjin, China; 2 Department of Spine Surgery, Tianjin Union Medical Center, The First Affiliated Hospital of Nankai University, Tianjin, China; 3 Tianjin Institute of Spinal Surgery, Tianjin, China; 4 People’s Hospital of Jimo, Qingdao, China; 5 Tianjin Medical University, Tianjin, China; 6 Department of Orthopedics, Tianjin Medical University General Hospital, Tianjin, China

**Keywords:** functional outcome, pelvic compensation index, sagittal imbalance, short-segment lumbar fusion, spinopelvic parameters

## Abstract

**Objective:**

To evaluate whether postoperative spinopelvic compensation is associated with functional improvement in patients with residual sagittal imbalance after short-segment lumbar fusion.

**Methods:**

Clinical data from 104 patients with lumbar degenerative disease who underwent short-segment lumbar fusion and had sagittal vertical axis (SVA) >5 cm at the 2-year follow-up were retrospectively analyzed. Patients were divided into functional improvement (*n* = 64) and non-improvement (*n* = 40) groups according to whether their Oswestry Disability Index (ODI) improvement reached the minimal clinically important difference (MCID) of 12 points. Visual Analog Scale (VAS), Japanese Orthopaedic Association (JOA) score, ODI, and spinopelvic parameters, including pelvic incidence minus lumbar lordosis (PI-LL) and pelvic compensation index (PCI), were compared between groups. Logistic regression was used to examine postoperative parameters associated with ODI improvement. Baseline-adjusted separate models were supplemented by a joint PI-LL/PCI sensitivity model.

**Results:**

Interobserver ICCs ranged from 0.913 to 0.987. VAS and JOA scores improved significantly in both groups, while the 2-year ODI was significantly better in the functional improvement group (*P* < 0.001). Mean PI was approximately 51° and did not change significantly over time (mean change 0.04°; *P* = 0.563). The functional improvement group had higher postoperative lumbar lordosis, pelvic tilt, and PCI, as well as lower PI-LL. In the joint model, each 5° increase in postoperative PI-LL was associated with lower odds of reaching MCID (OR = 0.229, 95% CI 0.116–0.454; *P* < 0.001), while each 0.10 increase in PCI was associated with higher odds (OR = 2.643, 95% CI 1.291–5.411; *P* = 0.008). Joint-model VIFs ranged from 1.010 to 1.037. The apparent and optimism-corrected AUCs for the joint model were 0.866 and 0.845, respectively.

**Conclusion:**

Adequate spinopelvic compensation, reflected by higher PCI and lower postoperative PI-LL, was associated with favorable functional outcomes despite residual sagittal imbalance.

## 1 Introduction

During spinal degeneration, the degree of kyphosis generally tends to worsen progressively with age. Initially, the human body can compensate through pelvic retroversion and flexion of the hip and knee joints to maintain sagittal balance. However, as the disease advances, these compensatory mechanisms may gradually fail, eventually leading to sagittal imbalance—a condition characterized by abnormal spinal alignment in the sagittal plane that impairs posture and functional status. Clinically, this manifests as intractable back pain, lower extremity pain, and movement disorders. In severe cases, neurological damage may occur, further affecting limb motor function. Numerous studies have confirmed that sagittal balance is closely associated with health-related quality of life (HRQOL). Among the various parameters used to quantify sagittal alignment, spinopelvic parameters have shown better correlation with clinical outcomes (1).

To improve clinical efficacy, Berjano proposed a scoliosis classification system designed to guide precise deformity correction surgery in patients with sagittal imbalance (2). However, the complication rate associated with corrective surgery for adult degenerative scoliosis (ADS) remains relatively high, ranging from approximately 16% to 80%. With ongoing advances in surgical techniques and concepts, clinical strategies have gradually shifted toward shorter fusion segments, avoidance of long-segment fixation, and minimization of osteotomy range. These adjustments have significantly reduced perioperative complication risks (3). The normal reference values for sagittal balance remain controversial. Clinically, an SVA <5 cm is generally regarded as the surgical target. However, Schwab et al. reported that the normal reference range is 2–3 cm, while Roussouly et al. found a mean SVA of 35 ± 19.4 mm (4, 5). These findings suggest that not all patients with sagittal imbalance (SVA >5 cm) require long-segment deformity correction surgery. In such patients, sagittal imbalance may simply be a clinical accompanying phenomenon—such as neurogenic claudication, radiating pain, or instability-related pain—that originates from local degenerative lesions including spinal stenosis and segmental instability. In recent years, the concept of “functional sagittal balance” has gained increasing attention (6). Lafage et al. pointed out that, compared with theoretical normal values, surgical correction goals in elderly patients should focus more on clinical efficacy than on merely correcting radiological parameters, as postoperative compensatory mechanisms may be impaired in this population (7). Therefore, this study focused on patients with sagittal imbalance (SVA >5 cm) after lumbar fusion surgery, with two aims: ① to retrospectively analyze and compare the clinical prognosis of patients with sagittal imbalance after short-segment lumbar fusion; and ② to identify relevant factors associated with clinical prognosis in these patients.

## 2 Materials and methods

### 2.1 General information

A retrospective analysis was performed on the clinical data of 104 patients who were admitted to our department between January 2019 and January 2024 for lumbar degenerative diseases, underwent short-segment lumbar fusion, and developed sagittal imbalance (SVA >5 cm) during postoperative follow-up. This study was conducted in accordance with the Declaration of Helsinki and approved by the Ethics Committee of Tianjin Union Medical Center (No.: 2023C42). Informed consent was waived due to the retrospective nature of the study.

### 2.2 Inclusion criteria

(1) Age >50 years and diagnosed with lumbar disc herniation or lumbar spinal stenosis; (2) Presenting with low back pain, lower extremity radiating pain, weakness, or intermittent claudication; (3) No significant improvement after 3 months of conservative treatment; (4) Underwent short-segment lumbar fusion with ≤3 fusion segments; (5) SVA >5 cm at the 2-year postoperative follow-up.

### 2.3 Exclusion criteria

(1) Coronal Cobb angle >10°; (2) History of spinal tuberculosis, infection, tumor, or fracture; (3) History of spinal revision surgery; (4) Incomplete follow-up data; (5) Severe underlying diseases (e.g., cardiovascular, cerebrovascular, or pulmonary diseases) or coagulation disorders; (6) Neuromuscular diseases (e.g., Parkinson's disease, myopathy), systemic metabolic diseases, or long-term bed rest resulting in extremely limited activity.

### 2.4 Patient grouping

The MCID for ODI improvement after lumbar surgery was set at 12 points (8). Based on ODI improvement at the 2-year follow-up, patients were divided into the functional improvement group (ODI improvement ≥12 points) and the non-improvement group (ODI improvement <12 points).

### 2.5 Treatment methods

All surgeries were performed by a single spine surgeon with more than 10 years of experience.

Surgical indications: Intractable low back pain lasting >6 months with poor response to conservative treatments (e.g., medication, physical therapy, epidural injection); lumbar MRI showing lumbar degeneration involving fewer than 3 segments, severe spinal stenosis with compression of the dural sac or nerve roots, severe degeneration of lumbar facet joints, or radiographic segmental instability on preoperative dynamic x-ray (9).

Surgical procedure: All patients were placed under general anesthesia in the prone position. Routine disinfection, draping, and sterile film application were performed. A posterior midline incision was made based on preoperative localization. The skin, subcutaneous tissue, and lumbodorsal fascia were incised; the supraspinous ligament was divided; and subperiosteal dissection of the bilateral laminae to the articular processes and exposure of the transverse processes were performed. Pedicle screws were inserted bilaterally, and C-arm fluoroscopy confirmed satisfactory screw positioning. Spinal canal and nerve root decompression was performed as needed. After disc-space preparation, an interbody fusion cage was implanted, connecting rods were installed, bone grafting was performed, a drainage tube was placed, and the wound was closed layer by layer.

### 2.6 Follow-up and outcome measures

#### 2.6.1 Clinical indicators

Clinical data were collected by two senior spine surgeons who were not involved in the index operation. These evaluators recorded baseline characteristics, perioperative data, complications, and clinical scores, including VAS scores for low back pain (VAS-B) and lower extremity pain (VAS-L), ODI, and JOA low back pain scores. The operating surgeon was not involved in outcome classification.

#### 2.6.2 Imaging indicators

All patients underwent preoperative and postoperative standing full-spine anteroposterior and lateral x-ray examinations. Radiographic measurements were independently performed by two experienced spine surgeons using the Neusoft Imaging System V5.5.0 at Tianjin Union Medical Center (see Figure 1 for schematic diagram). The two radiographic evaluators were blinded to ODI MCID grouping. The mean of the two measurements was used for analysis; discrepancies were reviewed jointly only for data-quality checking. For reliability assessment, one observer repeated the measurements after a 2-week interval, and two-way random-effects absolute-agreement ICCs and Bland-Altman limits of agreement were calculated.

**Figure 1:**
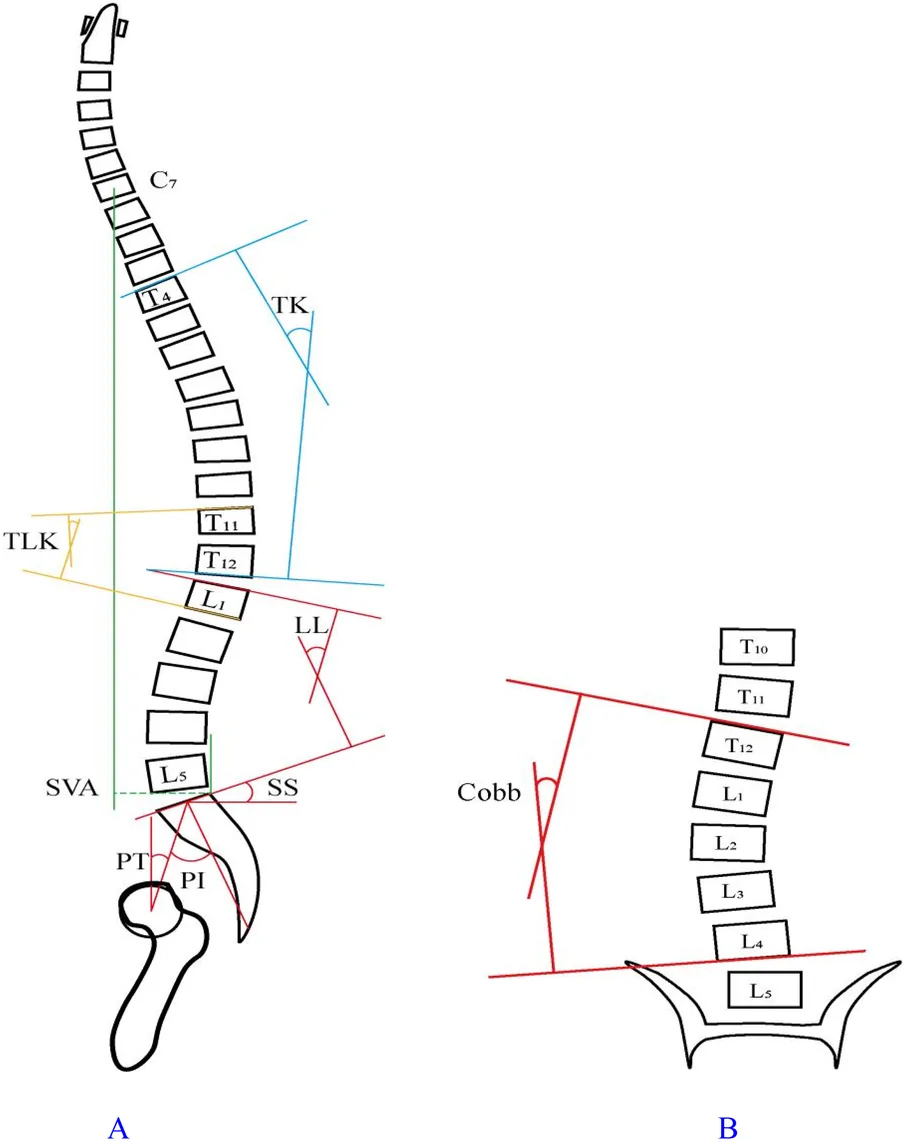
Schematic diagram of spinopelvic parameter measurement. **(A)** Sagittal view; **(B)** Coronal view. TK, Thoracic Kyphosis; LL, Lumbar Lordosis; SVA, Sagittal Vertical Axis; PT, Pelvic Tilt; PI, Pelvic Incidence; SS, Sacral Slope. The Cobb angle refers to the angle between the upper and lower endplates of the apical vertebrae in the coronal plane.

The spinopelvic parameters were measured as follows: ① Sagittal Vertical Axis (SVA): the horizontal distance between the C7 plumb line and the posterosuperior corner of S1 in the sagittal plane. ② Thoracic Kyphosis (TK): the angle formed by the upper endplate of T4 and the lower endplate of T12. ③ Lumbar Lordosis (LL): the angle formed by the upper endplate of L1 and the upper endplate of S1. ④ Pelvic Incidence (PI): the angle between the line perpendicular to the sacral endplate at its midpoint and the line connecting this midpoint to the femoral head axis. ⑤ Pelvic Tilt (PT): the angle between the vertical line and the line connecting the midpoint of the sacral endplate to the femoral head axis. ⑥ Sacral Slope (SS): the angle between the sacral endplate and the horizontal line. ⑦ Coronal Cobb Angle: the angle formed by the upper endplate of the upper vertebral body and the lower endplate of the lower vertebral body in the coronal scoliotic vertebrae. The geometric relationship PI = PT + SS was checked during data cleaning. The pelvic compensation index (PCI) was calculated as PT/PI, and PI-LL was calculated as PI minus LL.

### 2.7 Statistical analysis

Statistical analyses were performed using SPSS 25.0. This retrospective study had its sample size re-estimated before analysis. Based on the primary outcome (ODI), with *α* = 0.05 (two-tailed) and a statistical power of 95%, the current sample size was deemed to have sufficient power. The Kolmogorov–Smirnov test was used to assess normality of all samples. Normally distributed continuous data were expressed as mean ± standard deviation. Between-group comparisons were performed using independent samples t-tests; within-group pre- and postoperative comparisons used paired t-tests; categorical data were compared using chi-square or Fisher's exact tests. A *P*-value < 0.05 was considered statistically significant. Because this was a retrospective association analysis, postoperative variables were described as factors associated with ODI MCID achievement rather than as prospective predictors. MCID response was first examined in separate PI-LL and PCI logistic models, each adjusted for baseline ODI and age. A joint sensitivity model was then fitted including baseline ODI, age, PI-LL, and PCI to test whether PCI contributed information conditional on PI-LL. PT and PI were prespecified for structural exclusion because they are component variables of PCI and PI-LL. Predictors were scaled to clinically interpretable increments (5° for PI-LL, 0.10 for PCI, and 10 points for baseline ODI). Discrimination was summarized by AUC. The PCI cut-off was exploratory and derived from the current cohort; therefore, it requires external validation before clinical use.

## 3 Results

### 3.1 Baseline data

A total of 104 patients were included, consisting of 48 males and 56 females, with a mean age of 62.4 ± 7.1 years, mean BMI of 25.2 ± 3.4 kg/m^2^, and mean 2-year postoperative SVA of 6.4 cm. Among them, 64 patients were in the functional improvement group (27 males, 37 females; mean age 63.5 ± 7.7 years; mean BMI 25.5 ± 3.5 kg/m^2^; mean 2-year SVA 5.6 ± 1.7 cm), and 40 patients in the non-improvement group (18 males, 22 females; mean age 61.4 ± 6.9 years; mean BMI 24.9 ± 3.1 kg/m^2^; mean 2-year SVA 6.1 ± 2.0 cm). No significant differences were observed between the two groups in age, gender, BMI, preoperative ASA score, postoperative SVA, operation time, intraoperative blood loss, intraoperative fluoroscopy times, hospital costs, or hospital stay (*P* > 0.05) (Tables 1, 2). The K-S test results (sig >0.05) indicated normal distribution of the recorded data.

**Table 1:** Comparison of baseline data between the two groups of patients.

Characteristics	Improved group (*n* = 64)	Non-improved group (*n* = 40)	t/*χ*^2^	*P*-value
Age, years	63.5 ± 7.7	61.4 ± 6.9	1.407	0.162
Gender,n (male/female)	27/37	18/22	0.063	0.802
BMI, kg/m²	25.5 ± 3.5	24.9 ± 3.1	0.881	0.380
ASA physical status, *n* (Ⅱ/Ⅲ)	34/30	22/18	0.029	0.865

BMI, Body Mass Index; ASA, American Society of Anesthesiologists score; *P* < 0.05 was considered statistically significant.

**Table 2:** Comparison of perioperative clinical data between the two groups.

Characteristics	Improved group(*n* = 64)	Non-improved group(*n* = 40)	*t*	*P*-value
Operation time, min	146 ± 42	144 ± 44	0.231	0.818
Intraoperative blood loss, ml	310 ± 62	330 ± 68	1.527	0.130
Intraoperative fluoroscopy times, n	4.1 ± 1.9	3.8 ± 1.7	0.815	0.417
Length of hospital stay, days	8.4 ± 2.3	8.8 ± 2.7	0.793	0.430
Total hospitalization cost, CNY	64,617 ± 5499	66,148 ± 5,862	1.350	0.180

*P* < 0.05 was considered statistically significant.

### 3.2 Clinical follow-up

All patients completed 2 years of follow-up. Within-group comparisons showed that VAS-B, VAS-L, ODI, and JOA scores at 2 years were significantly improved compared with preoperative values in both groups (*P* < 0.05). Between-group comparison revealed a significant difference in ODI at 2 years (*P* < 0.001), while no significant differences were observed in VAS-B, VAS-L, or JOA scores (*P* > 0.05) (Table 3).

**Table 3:** Comparison of clinical parameters between the two groups before operation and at 2-year follow-up after operation.

Clinical parameter	Improved (*n* = 64)	Non-improved (*n* = 40)	*t*	*P*
VAS-B, preoperative	5.9 ± 1.3	5.7 ± 1.1	0.814	0.417
VAS-B, 2 years	2.2 ± 0.8	2.4 ± 0.9	1.174	0.243
VAS-L, preoperative	6.9 ± 1.9	6.7 ± 1.7	0.546	0.586
VAS-L, 2 years	1.7 ± 0.8	1.9 ± 1.0	1.106	0.271
JOA, preoperative	8.4 ± 2.1	8.2 ± 2.0	0.481	0.632
JOA, 2 years	22.9 ± 3.4	21.7 ± 3.1	1.815	0.072
ODI, preoperative	62.3 ± 19.4	58.5 ± 17.0	1.107	0.311
ODI, 2 years	35.4 ± 14.1	48.6 ± 13.5	4.748	<0.001

VAS-B, Visual Analogue Scale for low back pain; VAS-L, Visual Analogue Scale for lower extremity pain; JOA, Japanese Orthopaedic Association; ODI, Oswestry Disability Index; the Bonferroni correction was applied for multiple comparison test values, and *P* < 0.01 was considered statistically significant.

### 3.3 Spinopelvic parameters

Intra-group comparison showed that in the functional improvement group, the Cobb angle, SVA, LL, and SS decreased at 2 years compared with preoperative values, while PT, PCI, and PI-LL increased; no significant changes were observed in TK or PI. In the non-improvement group, the Cobb angle, SVA, LL, and SS also decreased, PT and PI-LL increased, PCI remained essentially unchanged, and TK and PI showed little variation.

Inter-group comparison revealed significant differences between the two groups at 2 years in LL, PT, SS, PCI, and PI-LL (*P* = 0.028, 0.005, 0.007, <0.001, and <0.001, respectively), whereas no significant differences were found in Cobb angle, SVA, TK, or PI (all *P* > 0.05). Mean PI was approximately 51° and did not change significantly from preoperative to postoperative assessment (mean change 0.04°; *P* = 0.563), consistent with PI being a fixed pelvic morphological parameter (Table 4).

**Table 4:** Comparison of spinal-pelvic parameters between the two groups of patients before surgery and 2 years after surgery.

Spinopelvic parameter	Improved (*n* = 64)	Non-improved (*n* = 40)	*t*	*P*
Cobb angle, preoperative (°)	7.4 ± 1.5	7.0 ± 1.3	1.552	0.121
Cobb angle, 2 years (°)	6.1 ± 1.7	5.7 ± 1.4	1.026	0.305
SVA, preoperative (cm)	7.3 ± 1.3	6.8 ± 1.5	1.793	0.073
SVA, 2 years (cm)	6.4 ± 0.7	6.5 ± 0.7	−0.702	0.483
TK, preoperative (°)	25.8 ± 11.9	27.3 ± 10.4	−0.671	0.502
TK, 2 years (°)	25.5 ± 12.2	27.2 ± 11.1	−0.711	0.477
LL, preoperative (°)	35.9 ± 7.5	37.3 ± 7.1	−0.903	0.366
LL, 2 years (°)	33.4 ± 7.8	30.0 ± 7.2	2.199	0.028
PI, preoperative (°)	50.4 ± 6.5	51.9 ± 5.7	−1.164	0.245
PI, 2 years (°)	50.4 ± 6.5	52.0 ± 5.6	−1.254	0.210
PI-LL, preoperative (°)	14.5 ± 5.0	14.6 ± 5.6	−0.121	0.903
PI-LL, 2 years (°)	17.1 ± 4.9	22.0 ± 4.5	−5.124	<0.001
PT, preoperative (°)	20.5 ± 4.5	20.7 ± 3.7	−0.286	0.775
PT, 2 years (°)	24.8 ± 3.7	22.8 ± 3.1	2.811	0.005
SS, preoperative (°)	29.9 ± 6.9	31.2 ± 6.6	−0.897	0.370
SS, 2 years (°)	25.6 ± 7.4	29.2 ± 4.9	−2.691	0.007
PCI, preoperative	0.4 ± 0.1	0.4 ± 0.1	0.373	0.709
PCI, 2 years	0.5 ± 0.1	0.4 ± 0.1	3.355	<0.001

Cobb angle refers to the angle between the upper and lower endplates of the apical vertebrae in the coronal plane; PCI is calculated as PT/PI; PI-LL = PI—LL. A *P* value < 0.05 was considered statistically significant.

Interobserver ICCs ranged from 0.913 (PT) to 0.987 (LL), and intraobserver ICCs ranged from 0.934 (PT) to 0.987 (LL).

### 3.4 Factors associated with ODI improvement reaching MCID

In the primary baseline-adjusted logistic regression model, each 5° increase in postoperative PI-LL was associated with lower odds of reaching the ODI MCID (OR = 0.235, 95% CI 0.123–0.449; *P* < 0.001), after adjustment for baseline ODI and age (Table 5).

**Table 5:** Baseline-adjusted logistic regression: postoperative PI-LL model.

Predictor	B	SE	OR	95% CI	*P*
Baseline ODI, per10	0.520	0.182	1.681	1.177–2.403	0.004
PI-LL, per5	−1.448	0.330	0.235	0.123–0.449	<0.001
Age, year	−0.085	0.037	0.918	0.853–0.988	0.022

LL denotes the lumbar lordosis angle. PI refers to the pelvic incidence angle. A difference of 0.05 was deemed statistically significant.

In the separate PI-LL model, each 5° increase in postoperative PI-LL was associated with lower odds of reaching MCID (OR = 0.235, 95% CI 0.123–0.449; *P* < 0.001). In the separate PCI model, each 0.10 increase in PCI was associated with higher odds (OR = 2.414, 95% CI 1.354–4.306; *P* = 0.003). In the joint sensitivity model, both associations persisted: OR = 0.229 per 5° PI-LL (95% CI 0.116–0.454; *P* < 0.001) and OR = 2.643 per 0.10 PCI (95% CI 1.291–5.411; *P* = 0.008), adjusted for baseline ODI and age. Joint-model VIFs ranged from 1.010 to 1.037, indicating no significant collinearity (Table 6).

**Table 6:** Baseline-adjusted PCI-only and joint PI-LL/PCI sensitivity models.

Model	Predictor	B	SE	OR	95% CI	*P*
PCI-only	Baseline ODI, per10	0.345	0.145	1.412	1.062–1.878	0.018
PCI-only	PCI, 2year,per01	0.881	0.295	2.414	1.354–4.306	0.003
PCI-only	Age, year	−0.024	0.032	0.976	0.916–1.040	0.450
Joint PI-LL + PCI	Baseline ODI, per10	0.512	0.190	1.668	1.149–2.422	0.007
Joint PI-LL + PCI	PI-LL,2year, per5	−1.474	0.349	0.229	0.116–0.454	<0.001
Joint PI-LL + PCI	PCI, 2year, per01	0.972	0.366	2.643	1.291–5.411	0.008
Joint PI-LL + PCI	Age, year	−0.075	0.039	0.928	0.859–1.002	0.056

LL denotes the lumbar lordosis angle, PI refers to the incidence angle of the pelvis. PCI represents pelvic compensatory function, defined as the PT/PI ratio. A *P* value of <0.05 was deemed statistically significant.

The PI-LL model had an apparent AUC of 0.822 and a bootstrap optimism-corrected AUC of 0.801. PCI alone had an AUC of 0.662. The joint model increased the apparent AUC to 0.866 (*Δ*AUC = 0.044) and had a bootstrap optimism-corrected AUC of 0.845. The exploratory PCI threshold was 0.530, with a sensitivity of 0.375 and specificity of 0.975 (Figure 2).

**Figure 2:**
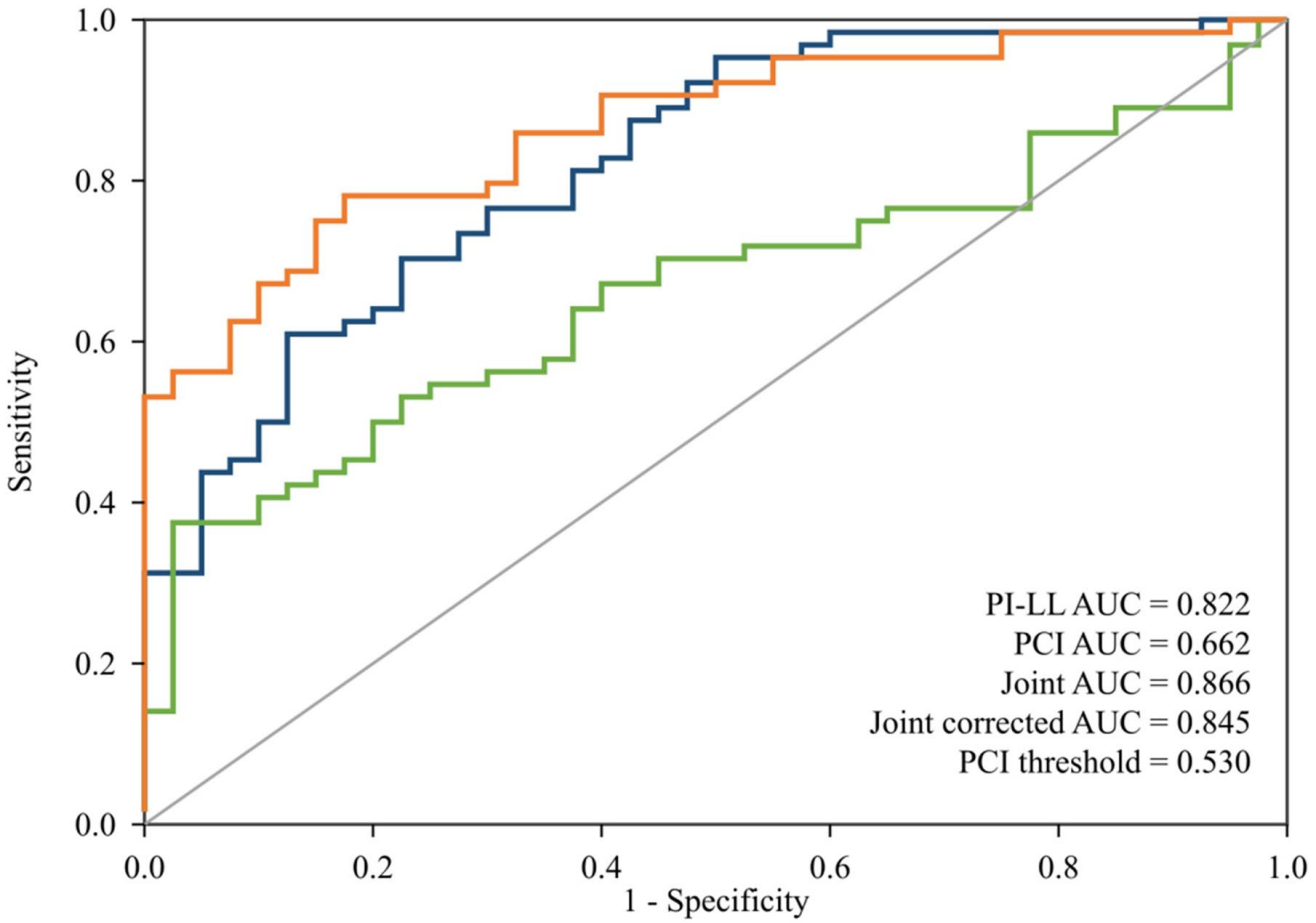
ROC curves for the PI-LL model, postoperative PCI, and the joint PI-LL/PCI model. Apparent AUCs were 0.822, 0.662, and 0.866, respectively; the joint bootstrap optimism-corrected AUC was 0.845. The exploratory PCI threshold was 0.530 (sensitivity 0.375, specificity 0.975).

### 3.5 Complications

No perioperative deaths occurred in either group. Complication details are presented in Table 7. The overall complication rate was 9.375% (6/64) in the functional improvement group and 10% (4/40) in the non-improvement group, with no significant difference between groups (*χ*^2^ = 0.011, *P* = 0.917).

**Table 7:** Comparison of postoperative complications between the two groups.

Complications	Improved group (*n* = 64)	Non-improved group (*n* = 40)	χ^2^	*P*
Cerebrospinal fluid leakage, n	3	1	—	—
Incision infection, n	1	1	—	—
Junctional fusion failure, n	0	1	—	—
Systemic complications, n	2 (pneumonia + heart failure)	1 (cerebral infarction)	—	—
Other complications, n	0	0	—	—
Total incidence, %	6/64 (9.375%)	4/40 (10.0%)	0.011	0.917

*P* < 0.05 was considered statistically significant.

### 3.6 Representative case

Representative preoperative and postoperative MRI, CT, and x-ray images meeting the inclusion criteria are shown in Figures 3, 4.

**Figure 3:**
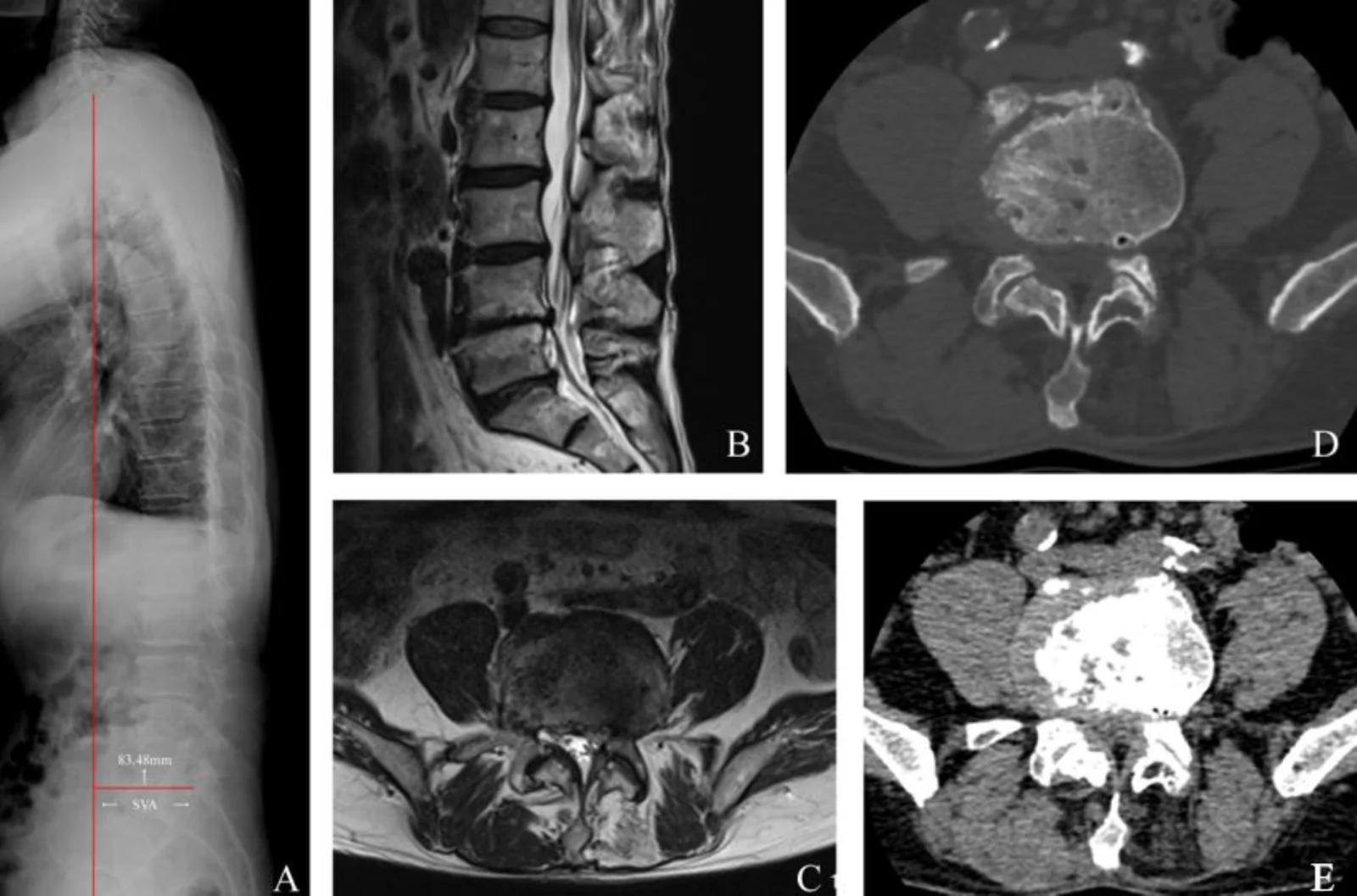
**(A)** preoperative full-spine lateral x-ray showing sagittal imbalance with an SVA of 83.48 mm. **(B–E)** Preoperative lumbar MRI and CT demonstrating intervertebral disc degeneration, compression and deformation of the dural sac, and stenosis of the lateral recesses and nerve root canals.

**Figure 4:**
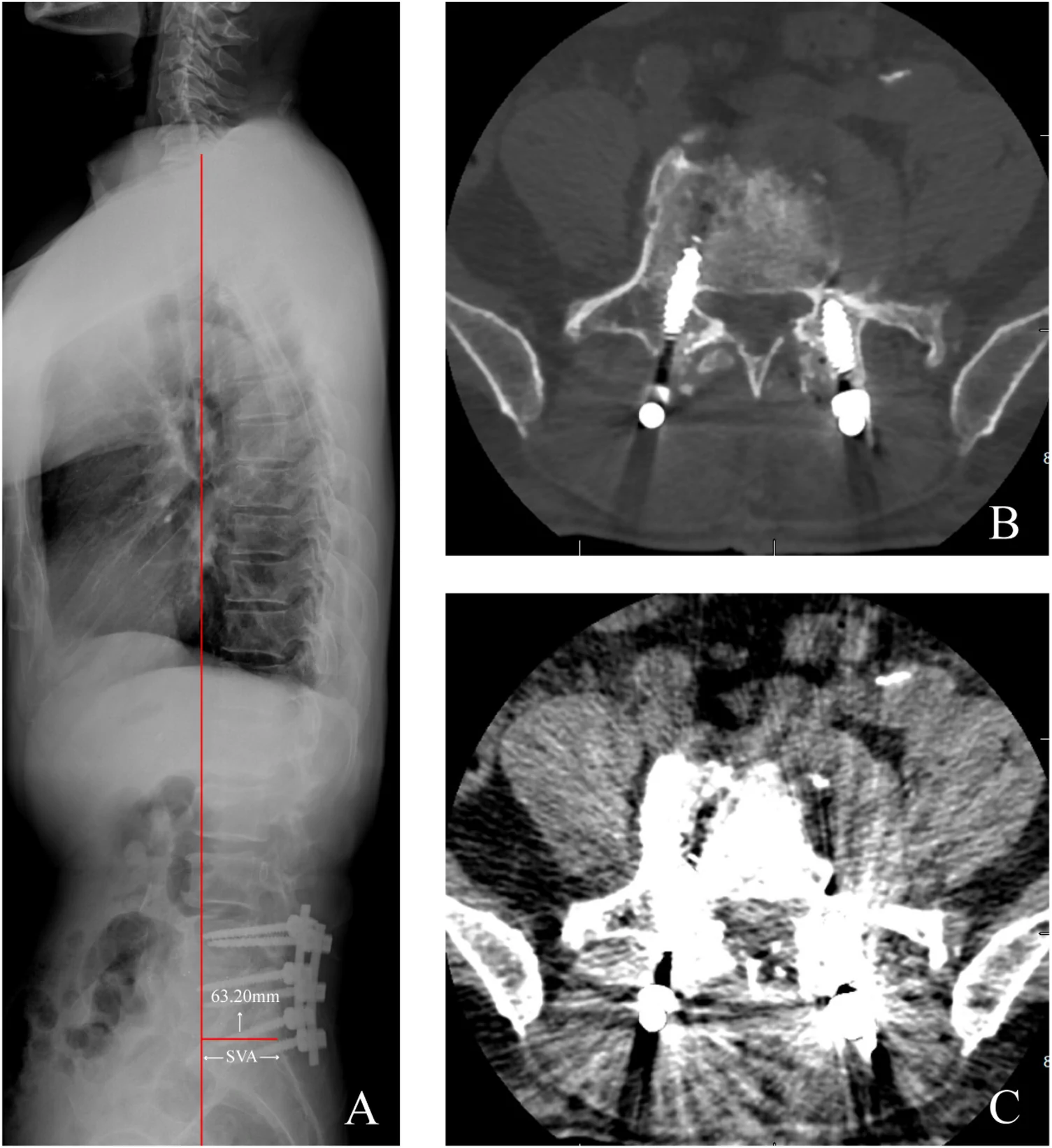
**(A)** postoperative full-spine lateral x-ray showing residual sagittal imbalance with an SVA of 63.20 mm. **(B,C)** Postoperative lumbar CT demonstrating satisfactory positioning of pedicle screws and interbody fusion cages, as well as adequate decompression of the spinal canal.

## 4 Discussion

### 4.1 Clinical efficacy

This study found that patients with postoperative residual sagittal imbalance (SVA >5 cm) could still achieve symptomatic improvement after short-segment lumbar fusion. Low back VAS, lower extremity VAS, and JOA scores improved in both groups, whereas ODI improvement differed significantly between groups. These findings suggest that residual sagittal imbalance does not uniformly preclude short-term symptomatic and functional benefit, and that factors beyond SVA may influence postoperative functional recovery.

The results showed that compared with preoperative values, VAS-B, VAS-L, and JOA scores improved significantly in both groups at 2 years (*P* < 0.05), with no significant intergroup differences (*P* > 0.05). ODI improved significantly in both groups postoperatively; 61.54% (64 patients) achieved the MCID for ODI improvement, while 38.46% (40 patients) did not. These findings indicate that in patients with SVA >5 cm after short-segment lumbar fusion, low back pain, lower extremity radiating pain, and neurological function can be significantly relieved regardless of whether ODI reaches MCID. However, a “dissociation phenomenon” was observed between pain relief and functional recovery (ODI): although pain relief was similar between groups, the degree of improvement in daily living activities differed significantly. A possible explanation is that VAS and JOA scores directly reflect adequate nerve decompression, while ODI comprehensively evaluates functional activities closely related to spinal biomechanics and environmental factors (e.g., standing, walking, sitting) (10). Even with complete neural decompression and significant pain relief, the mechanical abnormalities caused by sagittal imbalance are not fundamentally corrected, leading to functional impairment under long-term abnormal loading conditions (11). Thus, when evaluating clinical outcomes in patients with sagittal imbalance, functional disability indicators—like pain levels—are essential for accurately reflecting true postoperative efficacy.

### 4.2 Spinopelvic parameters and ODI improvement

Several postoperative spinopelvic parameters differed significantly between groups, particularly PT, PCI (PT/PI), and PI-LL. These findings suggest that postoperative spinopelvic compensation is closely associated with achieving ODI MCID. Despite residual sagittal imbalance (SVA >5 cm), some patients still achieved satisfactory functional recovery through effective spinopelvic compensation mechanisms (12).

Previous studies have shown that SVA >5 cm is an important radiographic marker of sagittal imbalance and is closely associated with postoperative HRQOL (13). However, the present study indicates that postoperative SVA alone may not fully explain functional prognosis. Although all patients had postoperative SVA >5 cm, 64 of 104 patients (61.54%) still achieved ODI MCID. This suggests that residual sagittal imbalance does not necessarily indicate poor functional recovery. Instead, the ability to maintain or restore spinopelvic compensation may help explain why some patients experience meaningful ODI improvement despite persistent sagittal malalignment. This finding is consistent with recent studies showing that sagittal alignment may improve after lumbar spinal stenosis surgery, and that residual sagittal imbalance should be interpreted together with clinical recovery rather than in isolation (14).Therefore, in elderly patients with degenerative lumbar disease—especially those with limited tolerance for extensive corrective surgery—clinical outcomes should not be evaluated solely based on whether SVA is corrected to <5 cm. The present results support a more comprehensive interpretation: even when SVA remains abnormal, favorable spinopelvic matching and compensatory pelvic adjustment may still contribute to postoperative functional recovery (15).

In this study, the functional improvement group demonstrated better postoperative spinopelvic alignment than the non-improvement group. Postoperative PI-LL was significantly lower in the improvement group (17.1° ± 6.8° vs. 22.0° ± 6.7°, *P* < 0.001), while postoperative PCI was significantly higher (0.50 ± 0.10 vs. 0.44 ± 0.06, *P* < 0.001). Postoperative PT was also higher in the improvement group (24.8° ± 4.5° vs. 22.8° ± 2.0°, *P* = 0.005). These results suggest that patients who achieved ODI improvement had relatively better pelvic compensation and less severe spinopelvic mismatch, even though their global sagittal alignment remained outside the conventional normal range. In other words, postoperative ODI improvement in patients with SVA >5 cm may depend more on the quality of spinopelvic compensation than on SVA correction alone.

Biomechanically, LL helps maintain the trunk's center of gravity over the pelvis and hip joints. When sagittal alignment is insufficient, the body may compensate through pelvic retroversion (reflected by increased PT) and hip extension to maintain an upright posture (16, 17). *n* the present study, postoperative LL was significantly greater in the functional improvement group than in the non-improvement group (33.4° ± 7.8° vs. 30.0° ± 7.2°, *P* = 0.028), while PI-LL was significantly smaller. This indicates that better restoration of LL and improved PI-LL matching may reduce the mechanical burden during standing and walking. Such coordinated compensation between the lumbar spine and pelvis may partly explain the greater ODI improvement observed in these patients (18). PCI may be particularly useful because it reflects pelvic compensation while accounting for individual pelvic morphology. PT alone is influenced by PI, and the same PT value may have different compensatory meanings in patients with different PI (19). By calculating PCI as PT/PI, the degree of pelvic retroversion can be interpreted relative to each patient's pelvic morphology (20). In this study, PCI was significantly higher in the functional improvement group, and ROC analysis showed that PCI had discriminative value for predicting ODI MCID achievement, with an AUC of 0.662 and an exploratory cut-off of 0.530. Although PCI alone had moderate predictive ability, the joint model combining PCI and PI-LL showed better discrimination, with an apparent AUC of 0.866 and a bootstrap-corrected AUC of 0.845. These findings suggest that PCI should not be interpreted in isolation, but rather together with PI-LL as part of a spinopelvic compensation framework.

Postoperative PI-LL was another important factor associated with functional improvement. PI-LL reflects the matching between pelvic morphology and lumbar lordosis, and PI-LL mismatch has been reported to be associated with low back pain and disability (21). In this study, patients who achieved ODI MCID had significantly smaller postoperative PI-LL than those who did not. Logistic regression further showed that lower postoperative PI-LL was independently associated with ODI improvement, even after adjustment for baseline ODI and age. These findings suggest that reducing PI-LL mismatch may be clinically meaningful in patients with residual SVA >5 cm. Therefore, even if SVA cannot be fully normalized, improving LL and reducing PI-LL may still provide functional benefit (22).

The clinical implication of this study is that the surgical goal for elderly patients with degenerative spinal disease should not be limited to aggressive correction of SVA. Long-segment corrective surgery aimed solely at achieving SVA <5 cm may increase surgical trauma and complication risk and may not be necessary for every patient. The present data suggest that in selected patients, maintaining or restoring spinopelvic compensation—particularly improving PI-LL and preserving adequate PCI—may allow meaningful ODI improvement even when postoperative SVA remains >5 cm. Therefore, surgical planning should emphasize individualized spinopelvic matching rather than simple radiographic normalization of SVA (23). However, because the exploratory PCI threshold showed high specificity but low sensitivity and was derived solely from this single-center cohort, it should be interpreted only as a supportive, hypothesis-generating reference. It must not be used directly as an independent clinical decision threshold without external validation.

### 4.3 Limitations

This study has several limitations. First, as a single-center retrospective analysis with a relatively small sample size, it is susceptible to selection bias and residual confounding. Second, potentially important determinants of pelvic compensation, including bone mineral density and paravertebral muscle mass, were not available and were therefore not included in the analyses. Third, the 2-year follow-up is insufficient to characterize long-term changes in compensatory status, durability of functional improvement, adjacent-segment degeneration, or other late complications. Fourth, the PCI cut-off of 0.530 was derived exploratorily in this cohort and has not been validated externally. It should be regarded only as a hypothesis-generating reference and must not be applied directly as a clinical decision threshold. Although bootstrap internal validation supported the apparent performance of the joint model, it cannot replace external validation. Future multicenter studies with larger samples, longer follow-up, and assessment of bone quality and paravertebral musculature are needed before PCI can be considered for clinical decision support. Finally, spinopelvic measurements require geometric and observer-reliability checks, and baseline ODI adjustment is necessary because outcome grouping is based on ODI change.

## 5 Conclusion

In conclusion, patients with predominant symptoms of low back pain, leg pain, and neurological dysfunction may achieve favorable short-term functional outcomes even when residual sagittal imbalance persists after short-segment lumbar fusion (SVA >5 cm). Patients with better functional recovery showed lower postoperative PI-LL and higher PCI, suggesting that spinopelvic matching and pelvic compensation may partly offset the adverse effects of residual SVA. Therefore, postoperative SVA should not be considered an absolute indicator of poor prognosis or the sole reason for aggressive long-segment correction. Surgical planning and postoperative evaluation should place greater emphasis on individualized spinopelvic compensation, especially the preservation or restoration of lumbar lordosis, pelvic compensation, and spinopelvic matching. These associations should be interpreted cautiously because of the retrospective single-center design, limited sample size, unmeasured confounding, and short follow-up. In particular, the PCI cut-off identified here is exploratory only and should not be used as a direct clinical decision threshold unless validated in independent external cohorts.

## Data Availability

The raw data supporting the conclusions of this article will be made available by the authors, without undue reservation.
